# Supplementary material for: Histone demethylase KDM2A is a selective vulnerability of cancers relying on alternative telomere maintenance
Source: Nat Commun. 2023 Mar 29;14:1756. doi: 10.1038/s41467-023-37480-2 (PMC10060224; doi:10.1038/s41467-023-37480-2)
Supplement: Supplementary file 2 — Reporting Summary [file 41467_2023_37480_MOESM2_ESM.pdf]

nature portfolio

Corresponding author(s): Hongwu Zheng

Last updated by author(s): 3/4/2023

Reporting Summary

Nature Portfolio wishes to improve the reproducibility of the work that we publish. This form provides structure for consistency and transparency in reporting. For further information on Nature Portfolio policies, see our Editorial Policies and the Editorial Policy Checklist.

Statistics

For all statistical analyses, confirm that the following items are present in the figure legend, table legend, main text, or Methods section.

n/a	Confirmed	
☐	☒	The exact sample size (*n*) for each experimental group/condition, given as a discrete number and unit of measurement
☐	☒	A statement on whether measurements were taken from distinct samples or whether the same sample was measured repeatedly
☐	☒	The statistical test(s) used AND whether they are one- or two-sided<br>*Only common tests should be described solely by name; describe more complex techniques in the Methods section.*
☐	☒	A description of all covariates tested
☒	☐	A description of any assumptions or corrections, such as tests of normality and adjustment for multiple comparisons
☐	☒	A full description of the statistical parameters including central tendency (e.g. means) or other basic estimates (e.g. regression coefficient) AND variation (e.g. standard deviation) or associated estimates of uncertainty (e.g. confidence intervals)
☐	☒	For null hypothesis testing, the test statistic (e.g. *F*, *t*, *r*) with confidence intervals, effect sizes, degrees of freedom and *P* value noted<br>*Give P values as exact values whenever suitable.*
☒	☐	For Bayesian analysis, information on the choice of priors and Markov chain Monte Carlo settings
☒	☐	For hierarchical and complex designs, identification of the appropriate level for tests and full reporting of outcomes
☒	☐	Estimates of effect sizes (e.g. Cohen's *d*, Pearson's *r*), indicating how they were calculated

Our web collection on statistics for biologists contains articles on many of the points above.

Software and code

Policy information about availability of computer code

Data collection	BD FACS DIVA 8.0.1
Data analysis	Live cell imaging movies were analyzed by Zeiss ZEN software (ver3.7, ZEISS); the images of terminal restriction fragment, C-circle and dot-blot were analyzed by Typhoon 9410 Imager (GE Healthcare) and processed with ImageQuant 5.2 software (Molecular Dynamics); cell cycle population analysis was conducted with FlowJo v5 software (FlowJo).

For manuscripts utilizing custom algorithms or software that are central to the research but not yet described in published literature, software must be made available to editors and reviewers. We strongly encourage code deposition in a community repository (e.g. GitHub). See the Nature Portfolio guidelines for submitting code & software for further information.

Data

Policy information about availability of data

All manuscripts must include a data availability statement. This statement should provide the following information, where applicable:

- Accession codes, unique identifiers, or web links for publicly available datasets
- A description of any restrictions on data availability
- For clinical datasets or third party data, please ensure that the statement adheres to our policy

The data that support the findings of this study are available within the article and its Supplementary Information files. All the uncropped western blots and raw data are provided as a Source data file.

Human research participants

Policy information about studies involving human research participants and Sex and Gender in Research.

Reporting on sex and gender	The patient-derived ATRX-mutant glioblastoma cell line pGBM6 was established from collected tumor specimens of a male patient .
Population characteristics	N/A
Recruitment	N/A
Ethics oversight	The patient-derived ATRX-mutant glioblastoma cell line pGBM6 was established from collected tumor specimens after obtaining written informed consent preoperatively and approved by the Institutional Reviewer Boards of the Southwest Hospital (KY2020147).

Note that full information on the approval of the study protocol must also be provided in the manuscript.

Field-specific reporting

Please select the one below that is the best fit for your research. If you are not sure, read the appropriate sections before making your selection.

[x] Life sciences [ ] Behavioural & social sciences [ ] Ecological, evolutionary & environmental sciences

For a reference copy of the document with all sections, see nature.com/documents/nr-reporting-summary-flat.pdf

Life sciences study design

All studies must disclose on these points even when the disclosure is negative.

Sample size	Cultured cells generally have little variability, and the coefficient of variation (CV) is often <0.1. Under this assumption, there will be 95% power to detect a 1.5-fold-change with a 2-sided alpha of 0.05 using a t-test and 3 biological replicates. Therefore, a minimum sample size of 3 was used for all the in vitro experiments. A sample size of 6 per group was used for the in vivo experiments. With 6 mice per each group, there will be 80% power at 5% significance level for two-sided test to detect odd of to achieve 8.3:1 ratio in average tumor volume between treatment and control groups.
Data exclusions	No data was excluded.
Replication	Each experiment was at least three times replicated.
Randomization	Mice were allocated to various treatment groups randomly (coin toss).
Blinding	Blinding was not applied since treatment groups were identified to the researchers.

Reporting for specific materials, systems and methods

We require information from authors about some types of materials, experimental systems and methods used in many studies. Here, indicate whether each material, system or method listed is relevant to your study. If you are not sure if a list item applies to your research, read the appropriate section before selecting a response.

Materials & experimental systems

n/a	Involved in the study	
[ ]	[x]	Antibodies
[ ]	[x]	Eukaryotic cell lines
[x]	[ ]	Palaeontology and archaeology
[ ]	[x]	Animals and other organisms
[x]	[ ]	Clinical data
[x]	[ ]	Dual use research of concern

Methods

n/a	Involved in the study	
[x]	[ ]	ChIP-seq
[ ]	[x]	Flow cytometry
[x]	[ ]	MRI-based neuroimaging

Antibodies

Antibodies used	For western blotting, Primary antibodies used were beta-ACTIN (ACTB) (1:5,000, #2228; Sigma), ATRX (1:1,000, sc-15408; Santa Cruz Biotechnology), FLAG (1:1,000, F1804; Sigma), H3 (1:2,000, ab1791; abcam), H3K36me2 (1:1,000, 2901; Cell Signaling), H3K36me3

	(1:2,000, 61101; Active Motif), KDM2A (1:1,000, A301-476A; Bethyl Laboratories), SENP6 (1:500, HPA024376; Sigma-Aldrich), SMC5 (1:2000, A300-236A; Bethyl Laboratories), and TUBULIN (1:2,000, ab15246; abcam). Secondary antibodies used were donkey anti-rabbit HRP (1:1,000, sc-2077; Santa Cruz Biotechnology), donkey anti-mouse HRP (1:1,000; sc-2096, Santa Cruz Biotechnology) and donkey anti-goat HRP (1:1,000, sc-2056, Santa Cruz Biotechnology).<br>For immunostaining, primary antibodies used were 53BP1 (1:500, IHC-00001; Bethyl Laboratories), 53BP1 (1:300, AF1877; R&D Systems), BLM (1:250, Cat# A300-110A; Bethyl Laboratories), FLAG (1:200, F1804; Sigma), γH2AX (1:2,000, A300-081A; Bethyl Laboratories), phospho-Histone H3 (Ser10) (1:200, #9701; Cell Signaling), PML (1:200, sc-966; Santa Cruz Biotechnology), PML (1:500, ab96051; abcam), SMC5 (1:400, A300-236A; Bethyl Laboratories), SUMO-2/3 (1:250, ab3742; abcam), and TRF1 (1:200, ab10579; abcam). Secondary antibodies conjugated to fluorophores were donkey anti-rabbit ALEXA488 (1:1,000, A21206, Thermo Fisher Scientific) or ALEXA647 (1:1,000, A31573, Thermo Fisher Scientific), donkey anti-mouse ALEXA488 (1:1,000, A21202, Thermo Fisher Scientific) or ALEXA647 (1:1,000, A31571, Thermo Fisher Scientific).<br>Antibodies used in ChIP assay were anti-FLAG (F1804, Sigma), anti-H3K36me2 (2901, Cell Signaling), and mouse IgG (sc2025, Santa Cruz Biotechnology).
Validation	All antibodies were commercial. Specificity and validation were provided by manufacturer's technical data sheets. The validation statements are available on the manufacture's website and their specificity was confirmed in literature. No further validation was performed.

Eukaryotic cell lines

Policy information about cell lines and Sex and Gender in Research

Cell line source(s)	The human cell lines A172 (male), HEK293T (female), HeLa (female), Hs792 (male), IMR90 (female), MCF-7 (female), NCI-H1299 (male), Saos2 (female), U118 (male), and U2OS (female) were obtained from ATCC. The IMR90-T, ALT#1, #2 and #3 cells were derived from large T-transformed IMR90 cells in this study. The patient-derived ATRX-mutant glioblastoma cell line pGBM6 (male) was established from collected tumor specimens after obtaining written informed consent preoperatively and approved by the Institutional Reviewer Boards of the Southwest Hospital. The human glioblastoma cell line LN464 (male) was kindly provided by F. Furnari (University of California, San Diego). The clonally derived KDM2A-knockout HeLa and LN464 cell lines, or ATRX-depleted IMR90-T and LN464-T cells were generated using lentiviruses produced in HEK293T cells with lentiCRISPR-v2 vectors containing KDM2A or ATRX targeting sgRNA and selected with blasticidin.
Authentication	Cell lines obtained from commercial source were not further authenticated. The LN464 cell line was authenticated by STR analysis.
Mycoplasma contamination	Cell lines were routinely tested for mycoplasma contamination. All cell lines were mycoplasma negative.
Commonly misidentified lines<br>(See ICLAC register)	None of the commonly misidentified cell lines were used.

Animals and other research organisms

Policy information about studies involving animals; ARRIVE guidelines recommended for reporting animal research, and Sex and Gender in Research

Laboratory animals	6-8 weeks NSG (NOD.Cg-PrkdcscidIl2rgtm1Wjl/SzJ) mice were purchased from Jackson laboratories. Mice were group housed (up to 5 per cage) in individually ventilated cages with ad libitum access to food and acidified water (pH 2.5 to 2.8) in a temperature (22.2± 0.5°C) and humidity (30–70%) controlled facility with 12:12-h light:dark cycle. The animal care and use program is accredited AAALAC. All animal experiments were approved by the Weill Cornell Institutional Animal Care and Use Committee.
Wild animals	The study does not include any wild animals.
Reporting on sex	Sex was not considered in this study.
Field-collected samples	The study does not include any such samples.
Ethics oversight	All of mouse use and procedures were approved by the Institutional Animal Care and Use Committee of the Weill Cornell Medicine.

Note that full information on the approval of the study protocol must also be provided in the manuscript.

Flow Cytometry

Plots

Confirm that:

- [x] The axis labels state the marker and fluorochrome used (e.g. CD4-FITC).
- [x] The axis scales are clearly visible. Include numbers along axes only for bottom left plot of group (a 'group' is an analysis of identical markers).
- [ ] All plots are contour plots with outliers or pseudocolor plots.
- [x] A numerical value for number of cells or percentage (with statistics) is provided.

Methodology

Sample preparation	Cells were permeabilized with cold 70% ethanol and resuspended in PBS containing PI (10 mg/mL) and RNAse A solution (10 ug/mL).
Instrument	Flow cytometry was performed using a LSRII flow cytometer (BD Biosciences).
Software	Cell cycle population analysis was conducted with FlowJo v5 software (FlowJo).
Cell population abundance	No sorting were performed.
Gating strategy	Single cells were gated based on FSC/SSC and cell cycle phases were determined by the FlowJo algorithm, as shown in FigS9a.

[x] Tick this box to confirm that a figure exemplifying the gating strategy is provided in the Supplementary Information.
