# Supplementary material for: Histone demethylase KDM2A is a selective vulnerability of cancers relying on alternative telomere maintenance
Source: Nat Commun. 2023 Mar 29;14:1756. doi: 10.1038/s41467-023-37480-2 (PMC10060224; doi:10.1038/s41467-023-37480-2)
Supplement: Supplementary file 4 — Description of Additional Supplementary Files [file 41467_2023_37480_MOESM4_ESM.pdf]

Title: Supplementary Movie 1.

Description: Time-lapse fluorescence live cell imaging depicting mitotic outcomes of control ALT#1 cells. GFP-H2B-expressing ALT#1 cells transduced with sgCtrl were synchronized in G2 with sequential thymidine and CDK1 inhibitor treatment before timed release into M phase. Time is shown as (hours: minutes: seconds) relative to the first image of the series. Scale bar, 100 μm. The square mark is used to denote the mitotic event(s).

Title: Supplementary Movie 2.

Description: Time-lapse fluorescence live cell imaging depicting mitotic outcomes of KDM2A-depleted ALT#1 cells. GFP-H2B-expressing ALT#1 cells transduced with sgK#1 were synchronized in G2 with sequential thymidine and CDK1 inhibitor treatment before timed release into M phase. Time is shown as (hours: minutes: seconds) relative to the first image of the series. Scale bar, 100 μm. The square mark is used to denote the mitotic event.

Title: Supplementary Movie 3.

Description: Time-lapse fluorescence live cell imaging depicting mitotic outcomes of control ALT#2 cells. GFP-H2B-expressing ALT#2 cells transduced with sgCtrl were synchronized in G2 with sequential thymidine and CDK1 inhibitor treatment before timed release into M phase. Time is shown as (hours: minutes: seconds) relative to the first image of the series. Scale bar, 100 μm. The square mark is used to denote the mitotic event(s).

Title: Supplementary Movie 4.

Description: Time-lapse fluorescence live cell imaging depicting mitotic outcomes of KDM2A-depleted ALT#2 cells. GFP-H2B-expressing ALT#2 cells transduced with sgK#1 were synchronized in G2 with sequential thymidine and CDK1 inhibitor treatment before timed release into M phase. Time is shown as (hours: minutes: seconds) relative to the first image of the series. Scale bar, 100 μm. The square mark is used to denote the mitotic event.

Title: Supplementary Movie 5.

Description: Time-lapse fluorescence live cell imaging depicting mitotic outcomes of control

IMR90-T cells. GFP-H2B-expressing IMR90-T cells transduced with sgCtrl were synchronized in G2 with sequential thymidine and CDK1 inhibitor treatment before timed release into M phase. Time is shown as (hours: minutes: seconds) relative to the first image of the series. Scale bar, 100 μm. The square mark is used to denote the mitotic event(s).

Title: Supplementary Movie 6.

Description: Time-lapse fluorescence live cell imaging depicting mitotic outcomes of KDM2A-depleted IMR90-T cells. GFP-H2B-expressing IMR90-T cells transduced with sgK#1 were synchronized in G2 with sequential thymidine and CDK1 inhibitor treatment before timed release into M phase. Time is shown as (hours: minutes: seconds) relative to the first image of the series. Scale bar, 100 μm. The square mark is used to denote the mitotic event.

Title: Supplementary Data 1.

Description: Gene dependency score (GDS) in the IMR90-T#1, IMR90-T#2, ALT#1, ALT#2, and ALT#3 cells. The GDS was calculated by averaging log2 fold-change (log2FC) of abundance (ratio of start to end point) of all sgRNAs targeting that gene.

Title: Supplementary Data 2.

Description: CRISPR-based KDM2A exon-tilling scan in IMR90-T, Saos2, ALT#1, and ALT#2 cells. The exon-tilling library comprises 492 sgRNAs targeting the entire KDM2A open reading frame. The data represent the fold changes of sgRNA abundance (ratio of start to end point) after 16 population doublings in culture.
