# Supplementary material for: Histone demethylase KDM2A is a selective vulnerability of cancers relying on alternative telomere maintenance
Source: Nat Commun. 2023 Mar 29;14:1756. doi: 10.1038/s41467-023-37480-2 (PMC10060224; doi:10.1038/s41467-023-37480-2)

Unedited gel for Figure 1

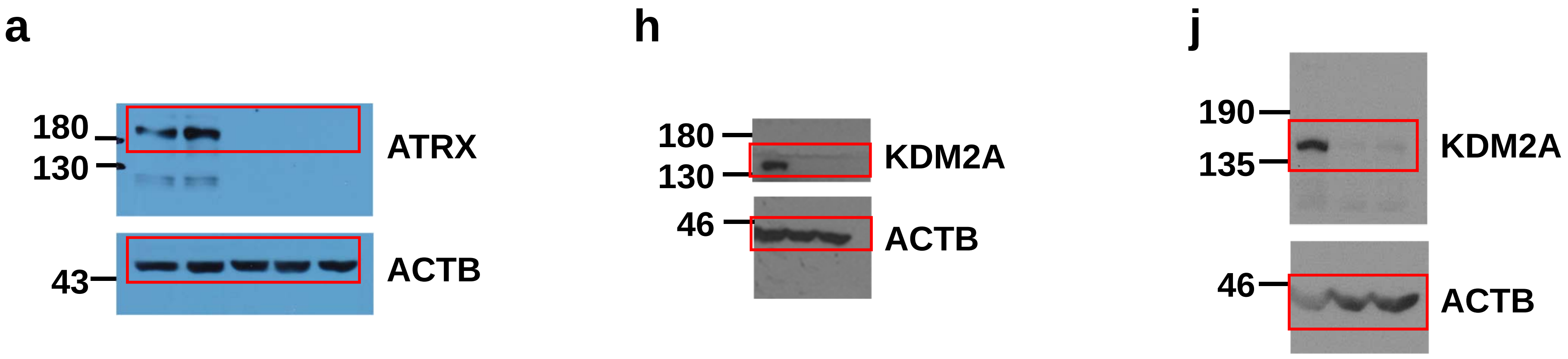

Unedited gel for Figure 2

e

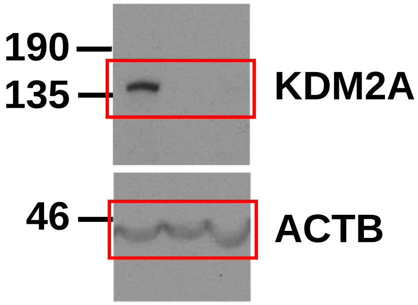

g

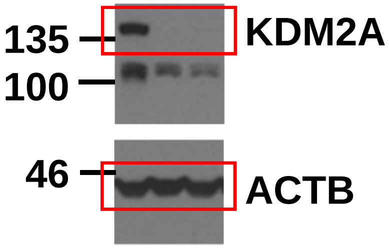

i

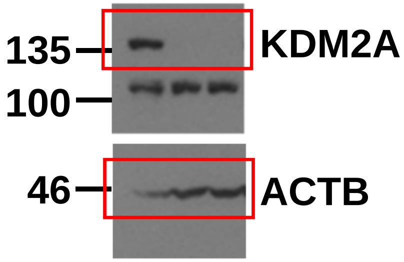

j

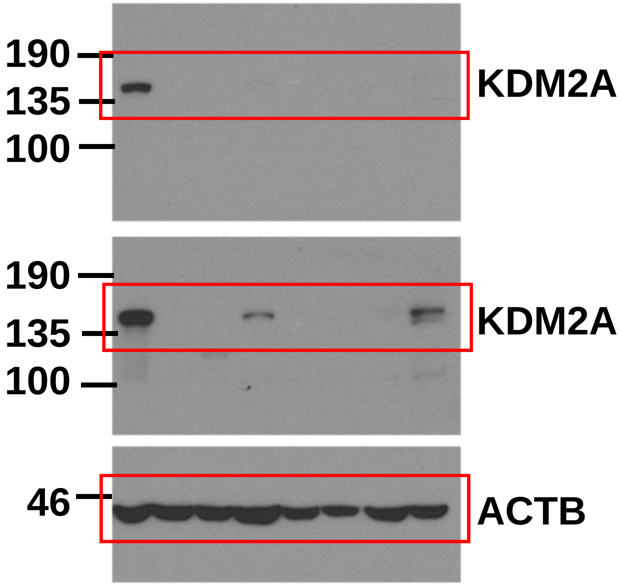

Unedited gel for Figure 3

d

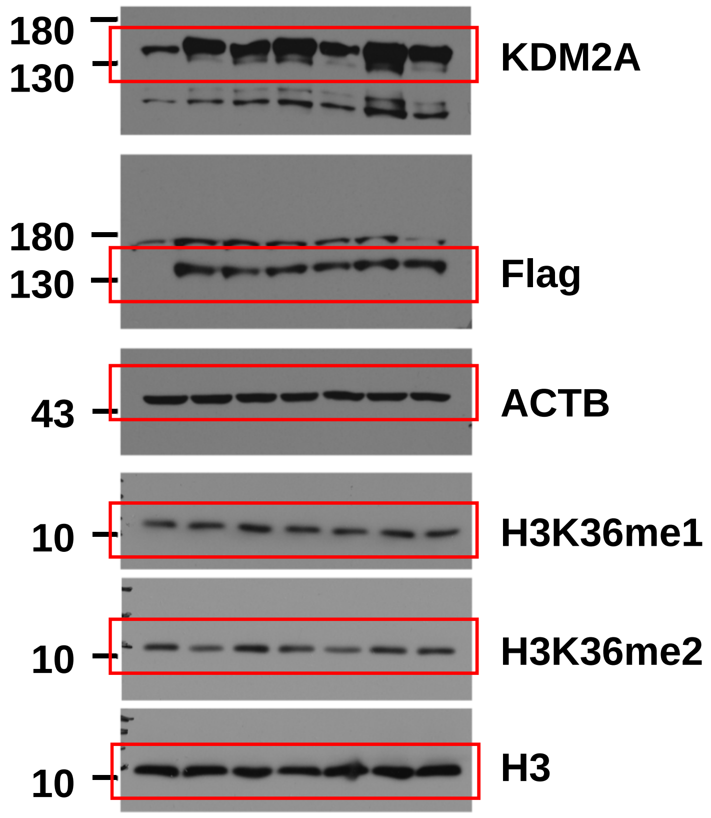

Unedited gel for Figure 4

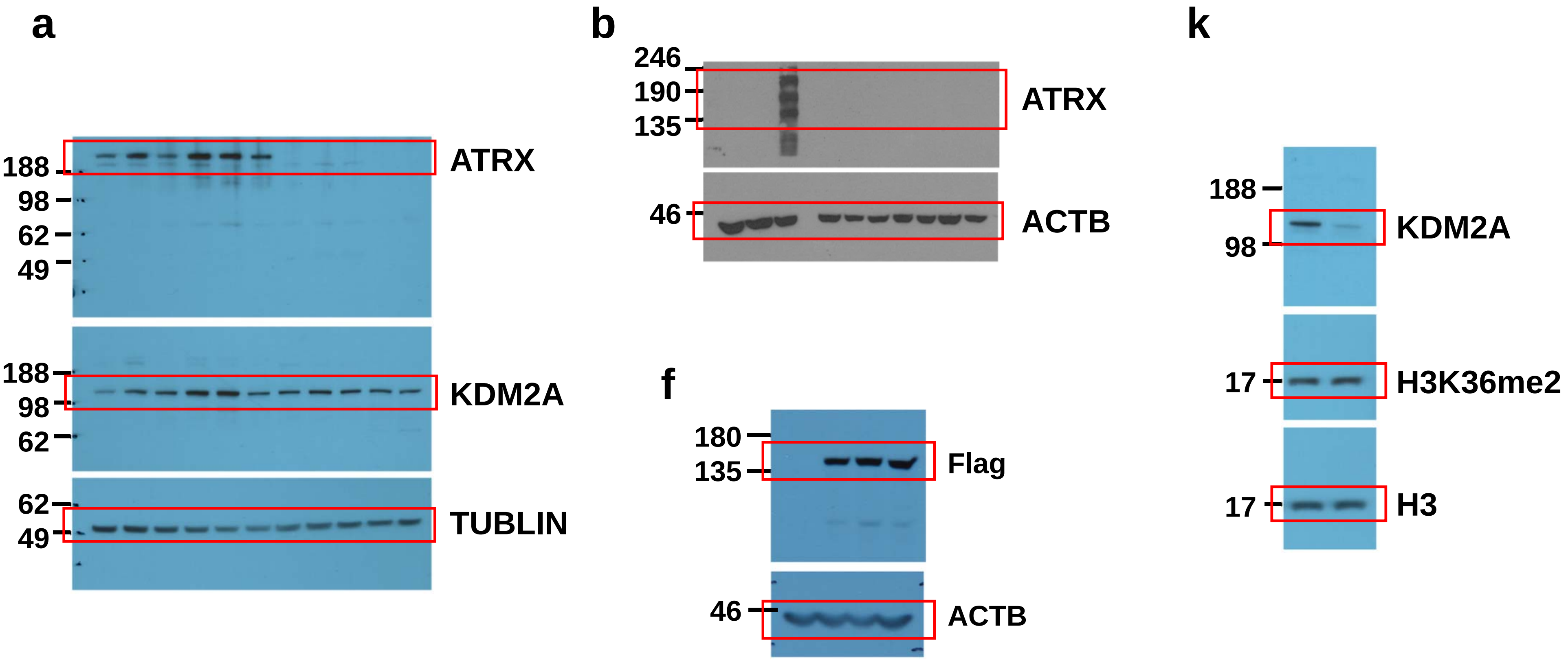

Unedited gel for Figure 6

c

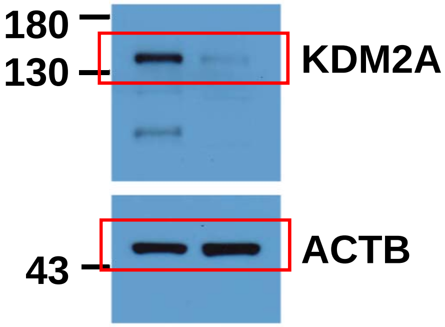

Unedited gel for Figure 7

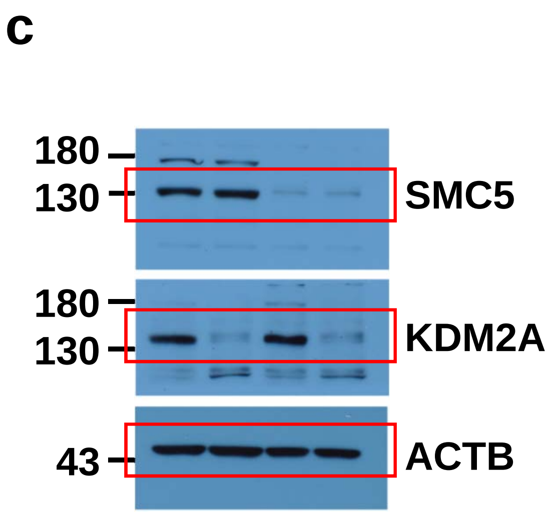

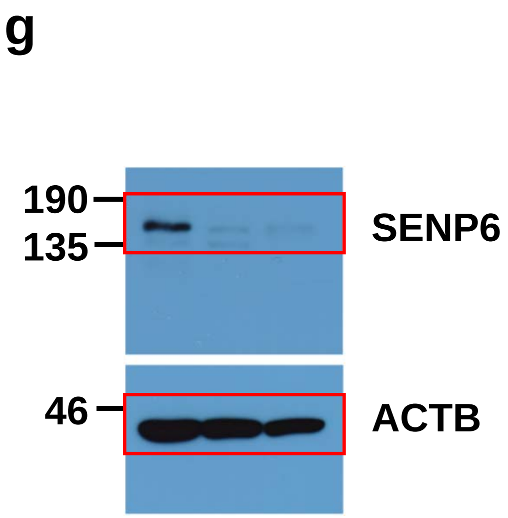

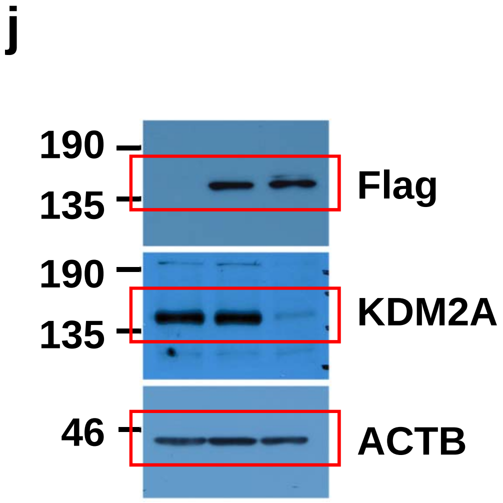

Supplement: Supplementary file 13 — Source Data [file 41467_2023_37480_MOESM13_ESM.zip › Source Data_Uncropped blots.pdf]
